# Cannabidiol and ∆9-Tetrahydrocannabinol in Endometriosis: A Literature Review on Therapeutic Applications and Mechanisms

**DOI:** 10.5935/1518-0557.20250168

**Published:** 2026

**Authors:** Joana Retzke Godoy, Júlia Prauchner de Castilhos, Fernanda Borsatto Caruso, Marta Ribeiro Hentschke

**Affiliations:** 1 Pontifical Catholic University of Rio Grande do Sul, Porto Alegre, RS, Brazil; 2 Federal University of Rio Grande do Sul, Porto Alegre, RS, Brazil

**Keywords:** endometriosis, cannabidiol, delta-9-tetrahydrocannabinol, infertility, pelvic pain

## Abstract

Endometriosis is a chronic, inflammatory, and multifactorial disease characterized by the presence of endometrial tissue outside the uterine cavity, often associated with debilitating symptoms. It affects approximately 10% of women of reproductive age and is also related to infertility. Endometriosis can be classified as peritoneal, ovarian, or deep endometriosis, with primary symptoms including chronic pelvic pain, dysmenorrhea, and dyspareunia. Diagnosis and treatment are challenging, with laparoscopy and biopsy of ectopic tissue being the gold standard. Cannabidiol (CBD) and ∆9-tetrahydrocannabinol (THC) are two major cannabinoids found in the *Cannabis sativa* plant, widely known for their medicinal properties. An experimental study conducted in rats demonstrated the anti-inflammatory, antioxidant, and antiangiogenic effects of intraperitoneal CBD use in the treatment of endometriosis. The objective of the present study was to conduct a literature review on the therapeutic potential of Cannabidiol (CBD) and ∆9-Tetrahydrocannabinol (THC) in the signs and symptoms of endometriosis. Research on PubMed, Embase, and Scopus platforms was conducted to determine the reproducibility and safety of treatment in humans, including dosage and administration route, as the current use is off-label.

## INTRODUCTION

Endometriosis is a chronic, inflammatory disease characterized by the presence of endometrial tissue outside the uterine cavity, affecting approximately 10% of women of reproductive age and often associated with infertility. Its main symptoms include chronic pelvic pain and dysmenorrhea. Treatment is challenging, and the use of cannabinoids, such as cannabidiol (CBD) and ∆9-tetrahydrocannabinol (THC), has shown promising therapeutic effects.

The hypothesis that the use of THC and CBD may bring benefits to patients diagnosed with endometriosis should be considered. Thus, the objective of the present study was to conduct a literature review on the therapeutic potential of Cannabidiol (CBD) and ∆9-Tetrahydrocannabinol (THC) in the signs and symptoms of endometriosis, investigating safety, dosage, and administration routes in humans.

## METHODS

A bibliographic review study was performed. For the research, the search platforms PubMed, Scopus, and Embase were used, with descriptors established through DeCS/MeSH for the first database and controlled vocabulary query for the other two.

In PubMed, the search strategy assembled was (Endometriosis[MeSH] OR Endometrioses OR Endometrioma OR Endometriomas) AND (Cannabidiol[MeSH] OR 1,3-Benzenediol, 2-(3-methyl-6-(1-methylethenyl)-2-cyclohexen-1-yl)-5-pentyl-, (1R-trans)- OR Epidiolex OR Cannabi* OR Resorcinols OR Terpenes OR “Dronabinol”[Mesh] OR THC OR Tetrahydrocannabinol OR “Tetrahydrocannabinol, Trans-Isomer” OR “Tetrahydrocannabinol, Trans Isomer” OR Marinol) AND (“Therapeutic Uses”[MeSH] OR “Uses, Therapeutic” OR “Therapeutic Use” OR “Use, Therapeutic” OR “Therapeutic Effects” OR “Effects, Therapeutic” OR “Therapeutic Effect” OR “Effect, Therapeutic”), with 103 results obtained.

In Scopus, the research was conducted using (Endometriosis OR Endometrioses OR Endometrioma OR Endometriomas OR “adenomyosis externa” OR “endometriosis externa”) AND (Cannabidiol OR Epidiolex OR Cannabi* OR Resorcinols OR Terpenes OR Dronabinol OR THC OR Tetrahydrocannabinol OR “Tetrahydrocannabinol, Trans-Isomer” OR “Tetrahydrocannabinol, Trans Isomer” OR Marinol OR adversa OR reduvo OR relivar OR syndros OR tetranabinex) AND (“Therapeutic Uses” OR “Uses, Therapeutic” OR “Therapeutic Use” OR “Use, Therapeutic” OR “Therapeutic Effects” OR “Effects, Therapeutic” OR “Therapeutic Effect” OR “Effect, Therapeutic” OR “drug treatment” OR “medicament therapy” OR “medicament treatment” OR “medicinal therapy” OR “medicinal treatment” OR “pharmaceutical therapy” OR “pharmaceutical treatment” OR “pharmaco-therapy” OR “pharmaco-treatment” OR “pharmacological therapy” OR “pharmacological treatment” OR pharmacotherapy OR pharmacotreatment OR “therapeutic uses” OR “therapy, drug” OR “therapy, pharmacological” OR “treatment, drug” OR “treatment, pharmacological”), with 426 final results.

Finally, in Embase, (‘adenomyosis externa’ OR ‘endometriosis externa’ OR ‘endometriosis’) AND (‘2 (6 isopropenyl 3 methylcyclohex 2 en 1 yl) 5 pentylbenzene 1, 3 diol’ OR ‘2 (6 isopropenyl 3 methylcyclohex 2 enyl) 5 pentylbenzene 1, 3 diol’ OR ‘2 [3 methyl 6 (1 methylethenyl) 2 cyclohexen 1 yl] 5 pentyl 1, 3 benzenediol’ OR ‘2 [3 methyl 6 (prop 1 en 2 yl) cyclohex 2 en 1 yl] 5 pentylbenzene 1, 3 diol’ OR ‘2 para mentha 1, 8 dien 3 yl 5 pentylresorcinol’ OR ‘5` methyl 4 pentyl 2` (prop 1 en 2 yl) 1`, 2`, 3`, 4` tetrahydrobiphenyl 2, 6 diol’ OR ‘a 1002 n5s’ OR ‘a1002n5s’ OR ‘btx 1204’ OR ‘btx 1308’ OR ‘btx 1503’ OR ‘btx 1702’ OR ‘btx 1801’ OR ‘btx1204’ OR ‘btx1308’ OR ‘btx1503’ OR ‘btx1702’ OR ‘btx1801’ OR ‘cardiolrx’ OR ‘epidiolex’ OR ‘epidyolex’ OR ‘gwp 42003’ OR ‘gwp 42003p’ OR ‘gwp42003’ OR ‘gwp42003p’ OR ‘nabidiolex’ OR ‘nantheia’ OR ‘oravexx’ OR ‘rad 011’ OR ‘rad011’ OR ‘trans cannabidiol’ OR ‘zygel’ OR ‘zyn 002’ OR ‘zyn002’ OR ‘cannabidiol’ OR ‘1 trans delta 9 tetrahydrocannabinol’ OR ‘3 pentyl 6, 6, 9 trimethyl 6a, 7, 8, 10a tetrahydro 6h dibenzo [b, d] pyran 1 ol’ OR ‘6, 6, 9 trimethyl 3 pentyl 6a, 7, 8, 10a tetrahydrobenzo [c] chromen 1 ol’ OR ‘adversa’ OR ‘bx 1’ OR ‘bx1’ OR ‘delta 1 3, 4 trans tetrahydrocannabinol’ OR ‘delta 1 tetrahydrocannabinol’ OR ‘delta 1 trans tetrahydrocannabinol’ OR ‘delta 1, 2 tetrahydrocannabinol’ OR ‘delta 9 tetrahydrocannabinol’ OR ‘delta 9 trans tetrahydrocannabinol’ OR ‘delta1 3, 4 trans tetrahydrocannabinol’ OR ‘delta1 cis tetrahydrocannabinol’ OR ‘delta1 tetrahydrocannabinol’ OR ‘delta1 thc’ OR ‘delta1 trans tetrahydrocannabinol’ OR ‘delta9 tetrahydro cannabinol’ OR ‘delta9 tetrahydrocannabinol’ OR ‘delta9 trans tetrahydrocannabinol’ OR ‘ea 1477’ OR ‘ea1477’ OR ‘l delta 9 trans tetrahydrocannabinol’ OR ‘levo delta 1 tetrahydrocannabinol’ OR ‘levo delta 9 tetrahydrocannabinol’ OR ‘marinol’ OR ‘ppp 002’ OR ‘ppp002’ OR ‘qcd 84924’ OR ‘reduvo’ OR ‘relivar’ OR ‘syndros’ OR ‘tetrahydrocannabinol 1 ene’ OR ‘tetrahydrocannabinol delta1’ OR ‘tetrahydrocannabinol delta9’ OR ‘tetranabinex’ OR ‘trans delta 9 tetrahydrocannabinol’ OR ‘u1 tetrahydrocannabinol’ OR ‘u9 tetrahydrocannabinol’ OR ‘dronabinol’) AND (‘drug treatment’ OR ‘medicament therapy’ OR ‘medicament treatment’ OR ‘medication’ OR ‘medicinal therapy’ OR ‘medicinal treatment’ OR ‘pharmaceutical therapy’ OR ‘pharmaceutical treatment’ OR ‘pharmaco-therapy’ OR ‘pharmaco-treatment’ OR ‘pharmacological therapy’ OR ‘pharmacological treatment’ OR ‘pharmacotherapy’ OR ‘pharmacotreatment’ OR ‘therapeutic uses’ OR ‘therapy, drug’ OR ‘therapy, pharmacological’ OR ‘treatment, drug’ OR ‘treatment, pharmacological’ OR ‘drug therapy’), pointing to 30 results.

All published articles were included without restrictions on date, species, or language. After the research, an analysis of all the articles found was conducted. The selection was made based on the titles and abstracts obtained in the searches. A total of 9 papers were analyzed and discussed.

### Endometriosis

Endometriosis is a chronic, inflammatory, and multifactorial disease, characterized by the presence of endometrial tissue outside the uterine cavity and very often associated with debilitating symptoms (Febrasgo, 2021). Affecting approximately 10% of women of reproductive age, it can present with adhesions and fibrosis or even hormonal and immunological changes, resulting in infertility (Tanbo & Fedorcsak, 2017). Studies indicate the presence of the disease in 25% to 50% of infertile women, with infertility occurring in 30% to 50% of women with the disease (Febrasgo, 2021).

Although the etiology of endometriosis remains unknown, several theories are considered on the subject. The primary one is described as the theory of retrograde menstruation, proposed in 1927 by John A. Sampson, which refers to the occurrence of menstrual flow in the opposite direction of the natural process, leading to the consequent intraperitoneal implantation and adhesion of endometrial cells (Sampson, 1927) (D’Hooghe & Debrock, 2002). It is understood that there must also be the influence of genetic, hormonal, or environmental factors (Nácul & Spritzer, 2010), as well as epigenetic factors (Chen *et al*., 2023). Endometriosis is an estrogen-dependent pathology, aggravated by the presence of the hormone estradiol leading to increased inflammation, pain, and other signs and symptoms (Bulun *et al*., 2012).

The most common symptoms involve chronic pelvic pain, dysmenorrhea, and dyspareunia, as well as tenesmus and hematochezia (Horne & Missmer, 2022). Furthermore, the disease can be classified as peritoneal, ovarian, and deep endometriosis, divisions that are respectively characterized by the presence of superficial implants on the peritoneum, superficial implants on the ovary (including cysts and endometriomas), and lesions of depth equal to or greater than 5 mm in the retroperitoneal space or on the wall of pelvic organs (Febrasgo, 2021).

One treatment alternative is the surgical removal of endometriotic foci through laparoscopy, which is also the gold standard for diagnosing the disease, aiming to identify and remove existing lesions (Mettler *et al*., 2014). Pelvic ultrasound and pelvic contrast-enhanced magnetic resonance imaging are exams that commonly support the suspicion of some cases of the disease, sometimes also providing the diagnosis (Rolla, 2019). Several pharmacological therapies are considered, such as the continuous use of progestogens (e.g., Dienogest), combined oral contraceptives (COCs), or even non-steroidal anti-inflammatory drugs (NSAIDs), aiming to treat signs and symptoms and prevent recurrence after surgical intervention. Drugs act by reducing the action of estradiol on the endometrium, leading to a decrease in inflammation and angiogenesis, with possible reduction of endometriotic lesions and even anovulation. Conversely, combined contraceptives may help by promoting an increase in progesterone (related to the reduction of ectopic endometriotic foci) and reducing ovarian estrogen production, in addition to anovulation. The main concern regarding the off-label use of COCs for endometriosis relates to the estrogen contained in the pill and its potential contribution to the progression of the disease. The class of NSAIDs primarily acts by reducing inflammation and prostaglandin production by inhibiting related enzymes, potentially influencing pain (Chen *et al*., 2023). Currently, depending on the patient’s condition and medical approach, opioids may be preferred over NSAIDs, which is concerning due to the drug class in question becoming a source of addiction, reinforcing the need for alternative pathways (Allam *et al*., 2022).

The choice of treatment varies among patients, depending on their medical history and the severity of symptoms or even the desire to conceive during the period, sometimes requiring a combination of surgical and pharmacological intervention. It is important to note that patients may exhibit progesterone resistance, which can potentially impact the response to certain medications throughout the treatment of endometriosis. Factors associated with inflammation, oxidative stress, epigenetics, disease phenotypes, and congenital characteristics influence such a response (Donnez & Dolmans, 2021). Moreover, women of childbearing age who desire to conceive need to discontinue the use of hormonal therapies. Regarding cases requiring surgical intervention, there is a known risk of adhesions and fibrosis after surgery, that could lead to infertility due to tubal factor, and the risk of decreased ovarian reserve in cases of surgical reduction of ovarian endometrioma (Chen *et al*., 2023). There is generally a significant delay in diagnosis, which directly impacts the quality of life of affected patients (Horne & Missmer, 2022).

### Cannabidiol and *∆*9-Tetrahydrocannabinol

The cannabis plant, according to geographical and evolutionary studies, originated millions of years ago, and by 4000 BCE, it was already being exploited as a source of fibers and food, as well as serving medicinal purposes and in rituals. In the 19th century, the physician William Brooke O’Shaughnessy conducted the first study demonstrating the pharmacological and toxicological properties of the plant. He also noticed differences between those cultivated in India (C. *indica*) and Europe (C. *sativa*) and evaluated their use in some of his patients, where he discovered analgesic and muscle-relaxing properties and explored their application in palliative care. Subsequently, in the 20th century, there was the discovery of the endocannabinoid system, its receptors, and the endogenous molecules that act on it, which renewed scientific interest in the properties of the plant (O’Shaughnessy, 1839) (Pisanti & Bifulco, 2019).

The endocannabinoid system (ECS) is made up of endogenous ligands, G protein-coupled membrane receptors (GPCRs), and enzymes to degrade the ligands. To date, two G-protein couplings related to this system have been identified, as well as two endogenous ligands (arachidonoyl ethanolamide and 2-arachidonoyl glycerol). The ECS works through a mechanism in which the carrier protein transports endocannabinoids retrograde from postsynaptic cell membranes to bind to cannabinoid receptors present on the presynaptic membrane, where they are degraded by the FAAH or MAGL enzymes. The half-life of endocannabinoids is very short, as they modulate the release of neurotransmitters by inhibiting the influx of intracellular calcium and are quickly reabsorbed and catabolized. Until the latest research, two types of signaling in the ECS were recognized, phasic signals and tonic signals. The tonic signals are known as basal signaling, responsible for endocannabinoid tone, while the phasic signals are responsible for the change in eCBs levels over time (Silver, 2019).

Cannabinoids, substances responsible for activating cannabinoid receptors, can be derived from the plant (phytocannabinoids), synthetic, or endogenous (endocannabinoids). The main cannabinoid receptors, known as cannabinoid receptors 1 and 2 (CB_1_ and CB_2_, respectively), are coupled to G protein, and the tissue distribution of these receptors is related to the consequent effects of interactions (Klein, 2005). The endocannabinoid system, present in all animals (including vertebrates and invertebrates, but in different forms), was discovered after the structural knowledge of ∆9-tetrahydrocannabinol (THC). It is known that CB_1_ and CB_2_ receptors are related to various biological processes such as pain, anxiety, inflammation, immune function, metabolic regulation, and others. While CB_1_ is mainly located in the central nervous system, CB_2_ is found mainly in cells of the immune system, spleen, and tonsils. In humans, CB_1_ receptors are significantly absent in the brainstem or spinal cord, which ensures safety in relation to vital functions. It is also known that CB_2_ receptors modulate cytokine release (Silver, 2019).

The Cannabis sativa plant is known worldwide for its medicinal and psychoactive properties, with more than 500 identified compounds, including cannabinoids, flavonoids, terpenes, and fatty acids (Alves *et al*., 2020). Among the various cannabinoids present in the plant, cannabidiol (CBD) and THC (Urits *et al*., 2020) are noteworthy. Both have medicinal properties; however, unlike CBD, THC induces psychoactive effects (Reich *et al*., 1982). THC acts as a partial agonist of CB_1_ and CB_2_ receptors, with activation or blockade of their activity by other cannabinoids depending on their level of expression and binding efficiency. CBD exhibits inverse agonism to CB_2_ (Thomas *et al*., 2007) and acts as an antagonist to CB_1_ and CB_2_ receptor agonists. Although it expresses low affinity for the receptors, it can interact at low concentrations (Pertwee, 2008). There is also significant interaction of CBD with transient receptor potential vanilloid 1 and 2 (TRPV1 and TRPV2) receptors, a fact related to the pharmacodynamic properties of the substance. TRPV2 is associated with analgesia (Jîtcă *et al*., 2023).

The THC molecule was identified in 1964 and has well-known therapeutic properties, with a complex and dose-dependent effect (Pernoncini & Oliveira, 2014). The principal therapeutic effects are antiepileptic, antiemetic, antispasmodic, analgesic properties, etc. It can present adverse effects, such as influencing the ability to discriminate time intervals and memory, as well as inducing disconnected thoughts, hallucinatory experiences, and others (Carlini, 2004). In contrast, the first studies demonstrating the medicinal potential of CBD emerged in 1982, where a protective effect against THC-induced psychoses was demonstrated. Subsequent studies revealed effects such as anxiolytic, anticonvulsant, antiemetic, anti-inflammatory, antioxidant, immunosuppressive, antiproliferative, pro-apoptotic, antiangiogenic, and others (Pernoncini & Oliveira, 2014). In relation to chronic pain, a symptom that affects about 20% of the population, CBD is a great candidate for use in treatment, although its analgesic effects are not yet fully understood (Urits *et al*., 2020).

### Cannabidiol and *∆*9-Tetrahydrocannabinol in Endometriosis

Dmitrieva *et al*. (2010) reported one of the first published articles on the subject, involving the role of the endocannabinoid system in endometriosis. Through a model using rats, the expression of CB_1_ receptors in the innervation of endometriotic foci was identified, as well as the reduction of hyperalgesia associated with the disease by CB_1_ receptor agonists. The research group also indicated that there is higher expression of the CB_1_ receptor in endometriotic lesions compared to the healthy uterus of the same rats, facts that suggest that treatments aimed at activating these receptors (either directly through CB_1_ agonists or indirectly by increasing endocannabinoid levels) can be performed with low impact on uterine function (Dmitrieva *et al*., 2010).

In the same year, Leconte *et al.* evaluated the antiproliferative and antifibrotic effects *in vitro* and *in vivo* of a cannabinoid agonist (WIN 55212-2) in deep endometriosis. The *in vitro* model was conducted using primary cultures of endometrial and deep endometriotic cells extracted from patients with or without the disease, while the *in vivo* model was conducted in mice by implanting deep infiltrating endometriotic nodules removed from human patients. They evaluated the presence of CB_1_ and CB_2_ receptors by comparing eutopic endometrial cell lines and deep endometriotic lesions. The cell lines were treated with increasing concentrations of the cannabinoid agonist, and a dose-dependent reduction in cell proliferation was observed. Additionally, they demonstrated that the treatment did not exhibit cytotoxicity at the studied concentrations and that there was a reduction in the production of reactive oxygen species (ROS). Furthermore, the inhibition of smooth muscle alpha-actin (αSMA) expression is related to the antifibrotic properties of the tested substance. The results were reproduced in the *in vivo* model, and thus, the treatment was considered promising for further studies (Leconte *et al*., 2010).

Allam *et al*. (2022) studied the expression of CB_1_ and CB_2_ receptors in ovarian endometriotic lesions. They determined, through immunohistochemistry, immunoblotting, and gene expression studies, the intense presence mainly in epithelial cells of such lesions. They also demonstrated increased expression of these receptors in ovarian endometriotic lesions compared to surrounding stromal tissues. Finally, the study suggested investigating the use of CB_2_ receptor agonists in the treatment of inflammation and pain related to ovarian endometriosis, given their relationship with cellular processes (Allam *et al.*, 2022).

A study was conducted on the exposure to THC in female mice with induced endometriosis, evaluating the effects associated with nociception, cognition, and emotion. THC treatment reduced hypersensitivity in a dose-dependent manner, particularly at 2 mg/kg/day, without impairing memory or inducing anxiety-like behavior. Long-term treatment (32 days) inhibited the development of endometriotic cysts, with no changes observed in the uterine or eutopic endometrial areas. The findings suggest that THC may selectively target ectopic endometrial cells and could have potential therapeutic effects for managing endometriosis-related pain and inflammation (Escudero-Lara *et al*., 2020).

Genovese *et al*. (2022) studied the anti-inflammatory, antioxidant, and analgesic effects of CBD related to endometriosis through an *in vivo* model in rats. The animals were divided into two groups, then classified as donors and recipients. The donors received intraperitoneal injections of exogenous gonadotropin (PMSG) to induce estrogen levels and were subsequently euthanized to remove the uterus and dissect the extrauterine tissue. The recipients, on the other hand, received intraperitoneal injections with a solution containing the tissue, aiming to develop the lesion in the region. Among the recipients, they were divided into three groups, being in order: endometriosis group; endometriosis + CBD group; and sham group. The first one received vehicle (ethanol/Tween 80/0.9% saline (3:1:16)), the second group received 10 mg/kg of CBD, and the third group received an intraperitoneal injection of phosphate buffered saline (PBS) in the midventral area as a substitute for endometrial tissue. Behavioral, histological, enzyme-linked immunosorbent assays, and measurements of levels of various neurosensitizing and pro-inflammatory substances were performed. They observed anti-inflammatory and antiproliferative effects regarding the size of the lesion and peritoneal fluids, as well as a decrease in neurogenic inflammation and neurosensitizing mediators (Genovese *et al*., 2022).

Okten *et al*. (2023) also studied the effects of CBD in an animal model. The experiments used rats, which were divided into four groups (saline solution, CBD 5mg/kg, CBD 20mg/kg, and leuprolide acetate), all undergoing three laparotomies, with intervals of 21 days. The first involved the induction of endometriotic lesions with tissue transplanted from the animal itself. The second aimed to confirm the formation of the implants and measure them, allowing injections according to the previously determined distribution. Finally, the third surgery was performed to collect samples of peritoneal fluid, in addition to measuring and collecting the implants, followed by euthanasia of the animals and collection of intracardiac serum samples. In the group that received CBD at a lower concentration, significant reductions were observed in the areas of the implanted endometriotic lesions compared to the group treated with saline, as well as in the levels of total antioxidant status (TOS), oxidative stress index (OSI), interleukin-6 (IL-6), and tumor necrosis factor-alpha (TNF-α), along with an increase in total antioxidant status (TAS) (Okten *et al*., 2023).

In studies involving humans, the prevalence, tolerability, and self-reported efficacy of Cannabis in women with endometriosis in Australia were evaluated. A questionnaire was administered to female individuals between 18 and 45 years old who had a surgically confirmed diagnosis of the disease. Various symptoms were addressed, including anxiety, depression, fatigue, gastrointestinal problems, nausea and vomiting, dyspareunia, dysuria, and sleep, in addition to pelvic pain and its impacts. They also inquired about costs, frequency of use (25% - less than once a week; 12.5% - once a week; 18.75% - two to three times a week; 43.75% - daily or several times a day), adverse events (10.42% - reported occurrence; 89.58% - did not report occurrence), and routes of administration (50% - smoking; 11.9% - vaporization; 11.9% - oral/edible; 2.38% - rectal or vaginal oil; 23.8% - multiple). Among the participants, 56% of women using the substance reported a significant reduction in medication use. The main improvements noted by the participants were related to pelvic pain and sleep, anxiety, depression, nausea/vomiting, and gastrointestinal symptoms. Reported adverse events included drowsiness, anxiety, and tachycardia. Participants who reported the use of medicinal cannabis had higher scores for the impact of pelvic pain compared to those who did not use it. The proportions of THC/CBD in the products consumed by the participants were not evaluated, and the reasons why participants consumed cannabis and/or other treatments were not explored (Sinclair *et al*., 2020).

Sinclair *et al*. (2021) studied the effects of cannabis ingestion on pelvic pain associated with endometriosis and other associated symptoms. The study was a retrospective cohort analysis using electronic records from users of a Canadian app for monitoring medicinal cannabis use. Statistical analysis of the data was performed, and self-rated efficacy was defined based on the initial and final classifications of reported symptoms. Self-reported symptoms were classified as pain (pelvic pain and dysmenorrhea), gastrointestinal (pain and nausea), and mood (depression and low libido), while forms of administration were divided into oral (edible, oil, capsule, spray), topical (transdermal and topical), and inhaled (vaporized and smoked, puffs). The mean age obtained was 35 years, with administrations mainly pulmonary (inhaled) and use mainly related to pelvic pain. The doses used ranged from 9mg/mL to 1mg/mL, as well as the concentrations of THC and CBD, both according to the chosen route of administration (Sinclair *et al*., 2021).

Sinclair *et al*. (2022) studied the use of cannabis in the treatment of endometriosis, with a larger sample of patients. The eligible participants were those who had confirmed endometriosis and have a history of using cannabis or products based on it for signs and symptoms control in the preceding three months. They inquired about the symptoms present at that moment (most of them being fatigue, chronic pelvic pain, and intestinal symptoms) and the medications/treatments being used at the time and, also, the access to Cannabis. The cessation or significant reduction of other medications while using cannabis was reported. The reasons for using cannabis were since inadequate pain control with other medications, intolerable side effects of other medications, perception of effects on signs and symptoms during recreational use, recommendations from colleagues/support groups, medical recommendation, difficulty accessing other medications, difficulty accessing surgery, and delayed/canceled surgery due to the COVID-19 pandemic. A significant number of participants reported that they would continue to use medicinal cannabis because it provided better effects in terms of pain relief compared to other current treatments. When questioned about communication and discussion of the use with healthcare professionals, generally there was no knowledge about legal access routes and no communication to doctors due to concerns related to legal and social issues, which poses risks to individuals’ health due to possible pharmacodynamic interactions and systemic effects. The group considered it a public health issue due to the widespread use of illicit cannabis, as it does not provide guaranteed quality and standardization of necessary active cannabinoids for regulation, nor does it provide significant safety for consistent clinical reproducibility, and it carries risks of contamination (mold, heavy metals, bacteria, and pesticide residues) (Sinclair *et al*., 2022).

Furthermore, there are already some studies in the development phase registered at the National Library of Medicine, such as NCT05670353 (São Paulo, Brazil) and NCT04527003 (Pennsylvania, United States of America) (ClinicalTrials.gov, 2023).

## CONCLUSION

Based on the findings above, several studies already indicate the therapeutic potential of cannabinoids ∆9-tetrahydrocannabinol and cannabidiol in the treatment of chronic pelvic pain, inflammation, cell proliferation, and other signs and symptoms directly associated with endometriosis. Clinical trials are needed to confirm the efficacy and safety of treatment in humans, also evaluating side effects, dosage, route of administration, and others.
